# Do marginalized neighbourhoods have less healthy retail food environments? An analysis using Bayesian spatial latent factor and hurdle models

**DOI:** 10.1186/s12942-016-0060-x

**Published:** 2016-08-22

**Authors:** Hui Luan, Leia M. Minaker, Jane Law

**Affiliations:** 1 Faculty of Environment, School of Planning, University of Waterloo, 200 University Avenue West, Waterloo, ON Canada; 2 Propel Centre for Population Health Impact, University of Waterloo, 200 University Avenue West, Waterloo, ON Canada; 3 Faculty of Applied Health Sciences, School of Public Health and Health System, University of Waterloo, 200 University Avenue West, Waterloo, ON Canada

**Keywords:** Neighbourhood marginalization, Retail food environment, Bayesian analysis, Spatial hurdle model, Spatial latent factor model

## Abstract

**Background:**

Findings of whether marginalized neighbourhoods have less healthy retail food environments (RFE) are mixed across countries, in part because inconsistent approaches have been used to characterize RFE ‘healthfulness’ and marginalization, and researchers have used non-spatial statistical methods to respond to this ultimately spatial issue.

**Methods:**

This study uses in-store features to categorize healthy and less healthy food outlets. Bayesian spatial hierarchical models are applied to explore the association between marginalization dimensions and RFE healthfulness (i.e., relative healthy food access that modelled via a probability distribution) at various geographical scales. Marginalization dimensions are derived from a spatial latent factor model. Zero-inflation occurring at the walkable-distance scale is accounted for with a spatial hurdle model.

**Results:**

Neighbourhoods with higher residential instability, material deprivation, and population density are more likely to have access to healthy food outlets within a walkable distance from a binary ‘have’ or ‘not have’ access perspective. At the walkable distance scale however, materially deprived neighbourhoods are found to have less healthy RFE (lower relative healthy food access).

**Conclusion:**

Food intervention programs should be developed for striking the balance between healthy and less healthy food access in the study region as well as improving opportunities for residents to buy and consume foods consistent with dietary recommendations.

## Background

A growing body of literature has shown that neighbourhood retail food environment (RFE) has a role in shaping residents’ food shopping and consumption behaviours [1–5]. Identifying and modifying characteristics of neighbourhood RFE could therefore be an important step in promoting population-wide healthy eating and reducing diet-related chronic diseases. An extensively explored research question is whether the RFE is less healthy in marginalized^1^ neighbourhoods, wherein residents are more vulnerable to adverse health outcomes. The exploration is largely motivated by the deprivation amplification hypothesis, which postulates that residents living in deprived neighbourhoods tend to have fewer health-promoting resources such as healthy foods [6]. In light of Lytle’s conceptual model of eating behaviours [7], the more people are constrained by individual (e.g., disability) and social (e.g., income) factors, the more their eating behaviours are explained by the food environment. In other words, Lytle’s model posits that marginalized residents are particularly at risk of poor diet and subsequent nutrition-related chronic disease if they live in a less healthy RFE.

Nevertheless, findings in terms of the association between marginalization and RFE are mixed across countries. Studies from the US consistently indicate that neighbourhoods with lower income and higher proportions of minority residents have reduced healthy food access, but the evidence is weak in other developed countries including Canada [8–10]. These inconsistent findings in past studies do not conclusively answer the question of whether marginalized neighbourhoods have a less healthy RFE, in part because of limitations in the approaches used to characterize the ‘healthfulness’ of neighbourhood RFE and neighbourhood marginalization as well as deficiencies in the statistical methods used.

### Characterizing neighbourhood RFE healthfulness

The ‘healthfulness’ of the neighbourhood RFE has been characterized using numerous methods. For example, focusing on absolute densities or numbers of so-called healthy food outlets such as supermarkets represents a focus on a single dimension of the complex RFE and thus could be biased. As reported, densities of healthy and less healthy food outlets are positively correlated, indicating that a neighbourhood could simultaneously have high densities of healthy and less healthy food outlets [11]. Recent studies have attempted to characterize the RFE healthfulness using relative healthy food access metrics, such as the proportion of healthy food outlets of all accessible food outlets, see for example the modified RFE index [12].

These relative measures however, ignore in-store characteristics (i.e., the quality and price of available foods as well as in-store marketing) which could vary within outlet types. For instance, the literature has suggested variations in shelf-space devoted to fruits and vegetables or healthy eating options, which have been proven relevant to healthy eating, within the same outlet types across neighbourhoods [13, 14]. Moreover, using outlet types to categorize healthy and less healthy food outlets has the potential to misclassify outlets and exclude outlets (e.g., specialty food stores) whose category is undetermined with a dichotomous classification scheme [15]. Another limitation associated with crude proportions for estimating neighbourhood RFE healthfulness is its uncertainty. Two areas with the same crude proportions, say 0.5, but different total number of accessible food outlets, say 2 and 20, respectively, are regarded to have a RFE with the same level of healthfulness.

### Characterizing neighbourhood marginalization

Much of the extant research is also limited by inadequate characterizations of neighbourhood marginalization. Most studies, in particular those in the US, have explored the association between individual socio-demographic and/or socio-economic indicators (i.e., proportions of low-income and minority residents) and the neighbourhood RFE. These individual indicators represent but a small fraction of marginalization which is a multi-faceted construct. Hence, many previous conclusions regarding these associations are actually based on associations between an oversimplified metric of the neighbourhood RFE and specific indicators of marginalization rather than multi-dimensional marginalization. The literature suggests that representing an overall construct such as marginalization by selecting a particular facet of the construct could “reduce strength of the intended signal and thus underestimate its association with the outcome of interest” [16]. In addition, selecting individual socio-economic or socio-demographic indicators is problematic since they may correlate with another indicator belonging to the same marginalization dimension, such that it could act as a proxy of its related indicator in the regression analysis and consequently the association. While the regression analysis could include all marginalization indicators, the multicollinearity problem is likely to occur.

Alternatively, marginalization can be measured with a composite index [17]. For instance, Larsen and Gilliland [18] calculated deprivation for London, Ontario by adding the standardized scores of percentage of lone-parent families, prevalence of low income, percentage of low educational attainment, and percentage of unemployment. Such a composite index may also be subject to arbitrary inclusion of marginalization indicators. Compared with the London case, a study conducted in Montreal, QC, Canada [19] included an additional indicator, the percentage of recent immigrants in the past 5 years, to operationalize deprivation.

Another limitation of current composite marginalization indices is that the included indicators are unweighted, an approach that assumes each indicator contributes evenly to marginalization. This assumption is problematic given that population structures vary across neighbourhoods [20]. To weight each indicator, statistical approaches implemented in the frequentist framework such as principal component analysis and factor analysis have been applied to construct the composite indices [17, 21–23]. These approaches are flawed in presuming that indicators (and the associated constructs they purport to measure) in adjacent areas are independent, an assumption usually violated in spatial studies at a small-area level.

### Statistical methods in neighbourhood RFE studies

Methodologically, with few exceptions, most studies use non-spatial statistical approaches to analyse the association between neighbourhood RFE and marginalization. For example, non-spatial versions of ordinary least square (OLS) and poisson/negative binomial regression approaches have been applied to model the continuous (e.g., distance to the nearest food outlet) [24, 25] and discrete (e.g., count of accessible food outlets) [22, 24, 26] measures of neighbourhood RFE, respectively. Residuals from regression analyses could be spatially auto-correlated given that spatial dependence is likely to exist between RFE measures at small-area levels with adjacent areas having similar RFE, a phenomenon rooted in the understanding that socioeconomic processes occur systematically and spatially across metropolitan areas [27]. Ignoring spatial autocorrelation renders conclusions regarding the association potentially invalid. The mixed findings in the literature could also be partly attributed to this methodological limitation. A recent meta-analysis of 54 papers revealed that although the spatial nature is widely acknowledged in RFE studies, very few adopted appropriate spatial statistical approaches [28].

Of the few studies that did use spatial approaches, Baker et al. [29] applied a spatial scan method to model the counts of fast-food restaurants and supermarkets in urban areas of St. Louis, Missouri. Their research found that mixed-race or white high-poverty communities and all-black communities regardless of poverty are less likely to have access to healthy foods compared to their predominantly white high-income counterparts. McKenzie [27] assessed neighbourhood disparities in supermarket access for Portland, Oregon region with a spatial error model. Findings revealed that in comparison to their counterparts in urban areas, neighbourhoods in suburban areas, either poor or non-poor, have longer travel distance and time to the nearest supermarket. Within suburban neighbourhoods however, the study found that deprivation was associated with shorter travel distance but longer travel time. Applying and comparing both spatial and non-spatial regression techniques, Wang et al. [30] analysed the relationship between spatial proximity to fresh food retailers and socioeconomic status in Saskatoon and Regina, Saskatchewan, Canada at the dissemination area level. In addition to identifying significant associations between healthy food access and socio-economic variables, their research reported that in comparison with spatial regression approaches, OLS overestimated the magnitude of the associations. Lamichhane et al. [31] analysed the relationship between access to supermarkets as well as fast-food outlets and neighbourhood characteristics with a Bayesian spatial Bernoulli model for the State of South Carolina at the census block group level. Several characteristics including income, housing value, and educational attainment were found to have a positive association with access to both supermarkets and fast-food outlets, whereas a negative association was identified for characteristics such as percentage of minority and population living under poverty after accounting for geographic location (e.g., urban, rural, etc.) and population density. Finally, Lamichhane et al. [32] applied a Bayesian spatio-temporal Poisson model to analyse the relationship between sociodemographic characteristics and densities of supermarkets and convenience stores for four US cities at the Census Tract level. Results indicated that poorer neighbourhoods have better access to both supermarkets and convenience stores after controlling for covariates including population density.

### Study objectives

To address the limitations in past studies, this research uses measures of the consumer nutrition environment (a Canadian adaptation of the widely-used NEMS-S [33] and the NEMS-R [34]) to classify “healthy” versus “less healthy” food outlets rather than assuming invariance in the consumer nutrition environment within outlet types.

Second, this study constructs four composite indices representing the four different marginalization dimensions for the study region, namely residential instability, material deprivation, dependency, and ethnic concentration, using a spatial latent factor model. A recent study reported that compared to its non-spatial counterpart, the spatial latent factor model provides more precise estimation for composite dimension scores, which thus enables more accurate assessment of the association between dimensions of neighbourhood environment and health outcomes [35]. Specifically, each marginalization dimension is derived from a number of relevant indicators which are theoretically informed and have been empirically validated [17]. These dimensions have been proven to be strongly and significantly associated with several public health outcomes derived from the nationally-generalizable Canadian Community Health Survey.

Finally, using Bayesian spatial hierarchical models, this research investigates whether marginalized neighbourhoods experience less healthy RFE. Healthfulness of neighbourhood RFE is represented as relative healthy food access and modelled via probability distributions rather than crude proportions of healthy food outlets. Various buffering sizes are used to characterize neighbourhood RFE, accounting for potential transportation modes. More details regarding the datasets and methodologies are given in the following sections.

## Study region and data

### Study region

**Fig. 1:**
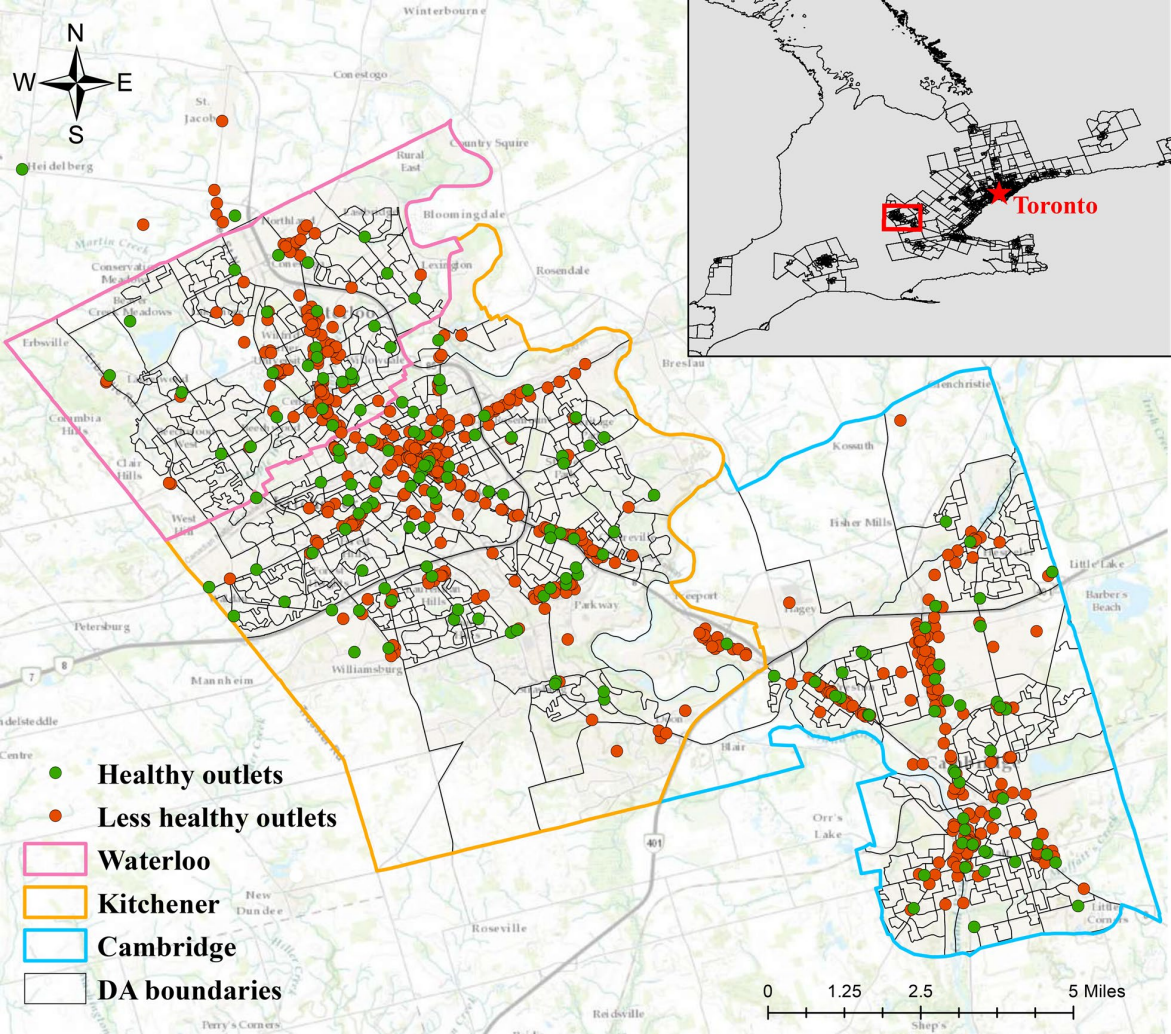
Boundaries of region of Waterloo and food outlet distributions, 2010

Our study was conducted in the Regional Municipality of Waterloo (Fig. 1), Ontario, specifically the cities of Waterloo, Kitchener, and Cambridge, which include 625 dissemination areas (DA). For reference, a DA is the smallest census area in Canada that covers the entire territory and follows roads and physical boundaries [36]. DAs are delineated such that the population size is generally between 400 and 700 [36]. The average population density in the study region was 3273.37/km^2^, ranging from 1.26 to 16,754.11/km^2^.

### Marginalization indicators

Guided by contemporary theories regarding marginalization in Canadian societies [37, 38] and the selection of characteristics for constructing areal deprivation indices in previous studies [39–41], we followed Matheson et al.’s approach [17] to include 18 indicators from 2006 Canadian census that belong to four marginalization dimensions: residential instability, material deprivation, dependency, and ethnic concentration (Table 1). The inclusion of these indicators enables a comprehensive depiction of neighbourhood marginalisation which involves diversified social problems relevant to health. The hypothesized loading sign of the indicator and its corresponding marginalization dimension, which indicates the direction of correlation, is also presented. For example, percentage of living alone (R1) is assumed to be positively associated with residential instability, whereas percentage of dwellings that are owned (R6) is presumed to have a negative loading.

**Table 1:** Variables used to measure marginalization dimensions, with hypothesized sign of loadings

ID	Indicator	Hypothesized loading sign
Residential instability		
1	% of living alone (R1)	+
2	% of youth population aged 5–15 (R2)	−
3	Crowding: average number of persons per dwelling (R3)	−
4	% of multi-unit housing (R4)	+
5	% of the population that is married/common-law (R5)	−
6	% of dwellings that are owned (R6)	−
7	% of residential mobility (same house as 5 years ago) (R7)	+
Material deprivation		
8	% 25+ without certificate, diploma, or degree (M1)	+
9	% of lone-parent families (M2)	+
10	% of government transfer payment (M3)	+
11	% of unemployment 15+ (M4)	+
12	% of below low income cut-off (M5)	+
13	% of homes needing major repair (M6)	+
Dependency		
14	% of seniors (65+) (D1)	+
15	Dependency ratio [(0–14) + (65+)]/(15–64) (D2)	+
16	Labor force participation rate (aged 15+) (D3)	−
Ethnic concentration		
17	% of 5-year recent immigrants (E1)	+
18	% of visible minority (E2)	+

### Measures of neighbourhood RFE

Food stores and restaurants were categorized as healthy if their nutrition environment measures survey (NEMS-S or NEMS-R) score fell within the highest two quartiles. The NEMS-S [33] and NEMS-R [34] are inventory-type measures of food stores and restaurants, respectively, that score outlets according to the quality, relative affordability, availability, and marketing of foods and beverages that comprise a large proportion of caloric intake at the population level. Data collection methods employed in the current study have been reported in detail elsewhere [42, 43]. Briefly, the Region of Waterloo’s public health inspection database was used to identify food outlets, and systematic direct observation was used to identify additional outlets and remove non-existent food outlets within the three cities from the sampling frame. One of each chain convenience store, pharmacy and superstore, and each grocery store and independently-owned convenience store, pharmacy, and specialty store in the three cities were assessed using the NEMS-S adapted for Canada (n = 422 stores). One of each chain restaurant and each independently-owned restaurant was assessed using the NEMS-R (n = 912). NEMS food outlet scores ranged from 0 to 43 for food stores and from −11 to 37 for restaurants. Data were collected in 2010.

The number of accessible healthy and total food outlets within 1, 4, and 8 km network buffering zones were calculated from each DA’s centroid. The first cut-off represents a walkable distance (10–15 min) which has been widely used in Canadian studies [18, 19, 24, 44], while the second, which has been used in past research for the same study region [45], represents a 5-min driving distance and also represents accessibility for people who use alternative transportation modes such as bicycling and public transit. A third buffering size which approximately represents a 10-min driving distance, 8 km, is used for testing the sensitivity in terms of how the relationships change under the assumption that residents own cars. Descriptive statistics of accessible healthy and total food outlets are shown in Table 2.

**Table 2:** Descriptive statistics of accessible food outlets within 1, 4, and 8 km from DA centroids

Buffering size (km)	Food outlets	Mean	Min.	Max.	SD
1	Healthy	5.1	0	48	7.6
	Total	11.6	0	117	17.5
4	Healthy	82.1	2	208	51.4
	Total	178.6	3	478	119
8	Healthy	249.3	32	414	94.9
	Total	527.5	52	859	199.4

**Fig. 2:**
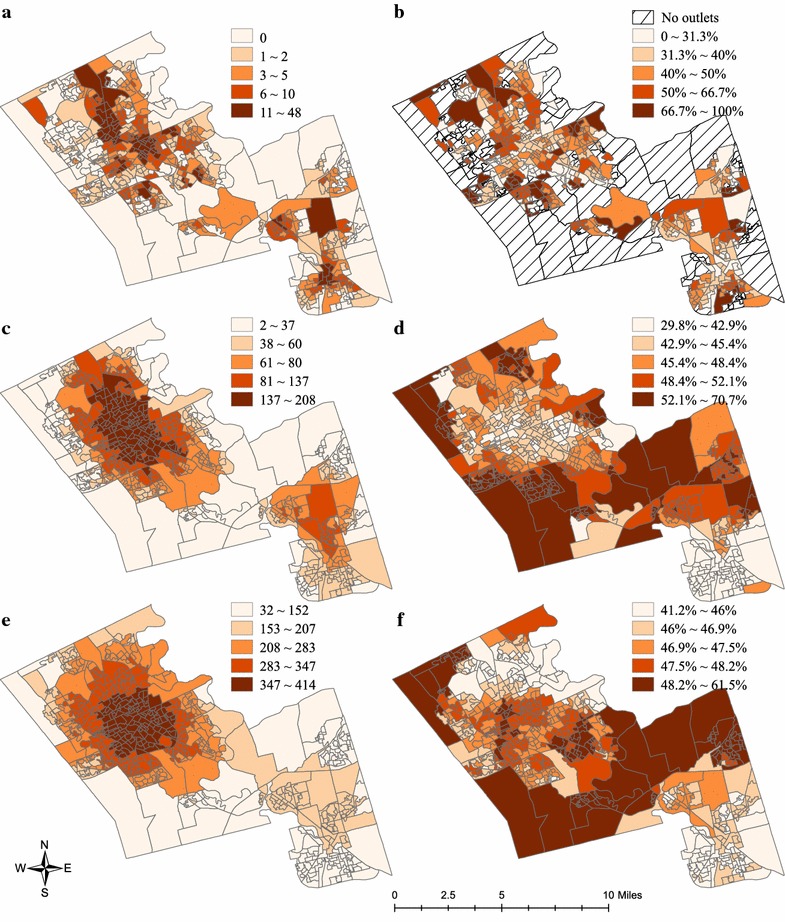
Quantile maps of count and crude proportion of healthy food outlets. **a** Count of healthy food outlets, 1 km. **b** Proportion of healthy food outlets, 1 km. **c** Count of healthy food outlets, 4 km. **d** Proportion of healthy food outlets, 4 km. **e** Count of healthy food outlets, 8 km. **f** Proportion of healthy food outlets, 8 km

The count and crude proportion of accessible healthy food outlets are mapped in Fig. 2. Areas without access to food outlets within a walkable distance are highlighted using hatch lines in Fig. 2b. Within 1 km, the central parts of the three cities have access to higher number of healthy food outlets. The spatial pattern becomes more distinct at the 4 and 8 km scales, with south Waterloo and north Kitchener having highest number of accessible healthy food outlets. In contrast, areas with higher crude proportions of healthy food outlets locate at peripheral parts of the cities, probably attributable to the relatively low number of total accessible food outlets. This pattern suggests that uncertainties exist in using the crude proportion to estimate the healthfulness of neighbourhood RFE.

## Methods

We use Moran’s I statistic to test spatial autocorrelation within each marginalization indicator and RFE measures including counts and crude proportions of healthy food outlets. A Moran’s I value approaching 1/−1 indicates strong positive/negative spatial autocorrelation, indicating that adjacent neighbourhoods have similar/dissimilar values of marginalization indicators and RFE. In contrast, a value equal to or close to zero suggests spatial randomness. In other words, values of marginalization indicators and RFE are randomly distributed over space. Spearman’s rank correlation analysis is applied to examine the correlations between indicators belonging to the same marginalization dimension. Below we detail the spatial latent factor model for constructing marginalization dimensions and spatial regression models for exploring the association between RFE healthfulness and marginalization dimensions as well as population density. All models are implemented in the Bayesian framework. For reference, Bayesian approaches combine prior knowledge and observed data to estimate posterior distributions of unknown parameters.

### Spatial latent factor model

Given that each marginalization indicator is theoretically linked to a specific dimension [17], the confirmatory rather than the exploratory factor model is used. Except for the dimension an indicator belongs to, the factor loadings of this indicator on other dimensions are set to zero. Similar approaches have been applied in Congdon [46–48]. Specifically, the normalized marginalization indicator j at area i (denoted as V_ij_) is assumed to follow a normal distribution with mean (α_j_ + δ_j_ × X_ni_) and variance $\upsigma_{\text{j}}^{2}$ [Model (1)], where X_ni_ is the nth marginalization dimension at area i (that V_ij_ belongs to), which is spatially structured (see Appendix 2 for more details); α_j_ is the intercept representing the average of indicator j over the study region; and δ_j_ is the factor loading of V_ij_ on X_ni_. For reference, the constructed factors X_1i_, X_2i_, X_3i_, and X_4i_ represent residential instability, material deprivation, dependency, and ethnic concentration, respectively.

1 $${\text{V}}_{\text{ij}} \sim{\text{Normal}}\left( {\upalpha_{\text{j}} +\updelta_{\text{j}} \times {\text{X}}_{\text{ni}} ,\upsigma_{\text{j}}^{2} } \right)$$

### Spatial regression models

#### Model for the 1 km dataset: spatial hurdle model

Considering that ~30 % of DAs (178 out of 625) had no access to healthy food outlets within a walkable distance and adjacent areas have similar healthy food access, we used a spatial hurdle model to analyse the 1 km dataset, accounting for the potential zero-inflation and spatial autocorrelation. Similar spatial hurdle models have been applied to model emergency department visits [49, 50] and adult mortality [51] with excess zeros. An alternative to the hurdle model for accounting for zero-inflation is the zero-inflated model [52], which assumes zeros arise from two sources—the “structural” zeros and “chance” zeros. The hurdle model is appropriate for this study because cases of zero accessibility are fully observed rather than latent—a DA either can or cannot access healthy food outlets within a walkable distance, and this access is not dependent on chance. Using a binomial hurdle model (more details given in Appendix 1), zero counts and positive counts are modelled via a Bernoulli distribution with probability parameter π_i_ and a truncated binomial distribution with probability parameter p_i_, respectively. Specifically, π_i_ represents the likelihood of a binary indicator—whether or not a DA has access to healthy food outlets, while p_i_ is the probability of a food outlet being healthy in DA_i_, which represents the prevalence of healthy food outlets (thus the healthfulness of neighbourhood RFE). Notably, p_i_ is equivalent to a modelled version of the relative healthy food access [45]. Compared with calculated or crude proportion of healthy food outlets, p_i_ is a more robust metric to reflect RFE healthfulness. Using a sampling distribution (i.e., binomial) to model empirical counts (e.g., the number of accessible healthy food outlets) that occur as proportions (i.e., the proportion of healthy food outlets), the uncertainty associated with crude proportions of healthy food outlets as shown in Fig. 2 can be accounted for by incorporating the sample size (i.e., the total number of accessible food outlets).

Logistic regression was further performed for π_i_ and p_i_ [Models (2), (3)], where α_1_ and α_2_ are intercepts for the Bernoulli and truncated binomial components and represent the average (logit) probability to access healthy food outlets and the (logit) average RFE healthfulness (or relative healthy food access) over the region, respectively. **X**^**T**^ is a 1 × 5 vector of covariates (with corresponding regression coefficient vectors **β**_**1**_ and **β**_**2**_ for Bernoulli and truncated binomial components, respectively). In particular, these coefficients represent the four marginalization dimensions (X_1i_, X_2i_, X_3i_, and X_4i_) estimated from Model (1) and population density—a major driving factor of food outlet distributions [53, 54]. The parameter vectors **u** (u_1i_ and u_2i_) and **s** (s_1i_ and s_2i_) are unstructured and spatial random effects (a.k.a., heterogeneity), respectively. These random effects are included to account for unmeasured covariates (spatial or non-spatial), overdispersion, and spatial autocorrelation [55].

2 $$\text{logit}(\uppi_{\mathrm{i}}) = \upalpha_{1} + {\text{X}}_{{\mathbf{i}}}^{{\mathbf{T}}} {\varvec{\upbeta}}_{{\mathbf{1}}} + \text{s}_{{1\text{i}}} + \text{u}_{{1\text{i}}}$$

3 $$\text{logit}(\hbox{p}_{\mathrm{i}}) = \upalpha_{2} + {\text{X}}_{{\mathbf{i}}}^{{\mathbf{T}}} {\varvec{\upbeta}}_{{\mathbf{2}}} + \text{s}_{{\text{2i}}} + \text{u}_{{\text{2i}}}$$

#### Model for the 4 and 8 km datasets: spatial binomial model

A regular spatial binomial model is used for the 4 and 8 km datasets because all DAs have access to healthy food outlets within the 4 and 8 km buffers. Specifically, the number of accessible healthy food outlet is assumed to follow a binomial distribution with probability parameter p_i_. Similarly, a logistic regression model is fitted for p_i_ [Model (4)]. Symbols in Model (4) refer to the same variables in Models (2) and (3).

4 $$\text{logit}(\hbox{p}_{\mathrm{i}}) = \upalpha + {\text{X}}_{{\mathbf{i}}}^{{\mathbf{T}}} {\varvec{\upbeta}} + \text{s}_{\text{i}} + \text{u}_{\text{i}}$$

### Model fit and implementation

Prior specifications are provided in Appendix 2. Models were implemented with the WinBUGS software [56]. The spatial latent factor model [Model (1)] was jointly implemented with spatial hurdle model [Models (2), (3)] and spatial binomial model [Model (4)], respectively, accounting for uncertainties associated with the constructed marginalization dimensions. Two parallel chains were fitted for the models, starting with diverging initial values. We checked model convergence by visually examining trace plots, history plots, autocorrelation plots, and Gelman–Rubin statistic plots. Model selection was based on the deviance information criterion (DIC) [57]. The best model is the one with lowest DIC. We ran each chain for 600,000 iterations, discarded the first 200,000 as burn-ins, and kept every 40th sample, resulting in a total of 20,000 samples for posterior estimates. Sensitivity analysis for prior specification was performed with alternative vague priors for parameters in the models. Similar results were obtained and DIC difference is smaller than 5, indicating that modelling results are insensitive to prior selections.

## Results

### Moran’s I analysis of marginalization indicators and RFE measures

Results of Moran’s I analysis for marginalization indicators are presented in Table 3. Most indicators are found significantly and spatially correlated with the exception of M4 (% of unemployment), D2 (dependency ratio), and E1 (% of 5-year recent immigrants), indicating the necessity to use spatial statistical approaches to construct the composite marginalization dimensions.

**Table 3:** Moran’s I test of marginalization indicators

ID	Indicator	Moran’s I
Residential instability		
1	% of living alone (R1)	0.537***
2	% of youth population aged 5–15 (R2)	0.467***
3	Crowding: average number of persons per dwelling (R3)	0.588***
4	% of multi-unit housing (R4)	0.371***
5	% of the population that is married/common-law (R5)	0.497***
6	% of dwellings that are owned (R6)	0.396***
7	% of residential mobility (same house as 5 years ago) (R7)	0.221***
Material deprivation		
8	% 25+ without certificate, diploma, or degree (M1)	0.488***
9	% of lone-parent families (M2)	0.11***
10	% of government transfer payment (M3)	0.384***
11	% of unemployment 15+ (M4)	0.066**
12	% of below low income cut-off (M5)	0.157***
13	% of homes needing major repair (M6)	0.362***
Dependency		
14	% of seniors (65+) (D1)	0.278***
15	Dependency ratio [(0–14) + (65+)]/(15–64) (D2)	0.038*
16	Labor force participation rate (aged 15+) (D3)	0.233***
Ethnic concentration		
17	% of 5-year recent immigrants (E1)	0.099***
18	% of visible minority (E2)	0.325***

p value: **** <0.001; ** <0.01; * <0.05

The smaller the p value, the less likely that the correlation occurs by chance

Table 4 shows results of Moran’s I test of count and crude proportions of healthy food outlets. All RFE measures at the three scales are significantly auto-correlated with high autocorrelation except the crude proportion at the 1 km scale, which has a moderate autocorrelation. This finding indicates that adjacent areas have similar absolute and relative healthy food access thus again demonstrates the necessity to apply spatial statistical approaches.

**Table 4:** Moran’s I test of count and crude proportions of healthy food outlets

Buffering size (km)	RFE measures	
	Count	Crude proportion
1	0.709***	0.295***
4	0.917***	0.805***
8	0.957***	0.701***

Crude proportion = (number of accessible healthy food outlets/total number of accessible food outlets) × 100

p value: *** <0.001; **< 0.01; * <0.05

### Bivariate correlation analysis of marginalization indicators

Results of bivariate analysis of marginalization indicators are shown in Table 5. As expected and consistent with previous findings [17], indicators belonging to the same marginalization dimension are significantly and highly or moderately correlated. Exceptions are R2 and R7, M1 and M4, and M4 and M6, which have significant but weak correlations.

**Table 5:** Bivariate correlation analysis between indicators belonging to the same marginalization dimension

Residential instability							
	R1	R2	R3	R4	R5	R6	R7
R1	1						
R2	−0.66***	1					
R3	−0.83***	0.78***	1				
R4	0.61***	−0.28***	−0.57***	1			
R5	−0.71***	0.55***	0.74***	−0.71***	1		
R6	−0.67***	0.39***	0.66***	−0.82***	0.78***	1	
R7	0.37***	−0.08*	−0.24***	0.56***	−0.35***	−0.52***	1

Material deprivation						
	M1	M2	M3	M4	M5	M6
M1	1					
M2	0.33***	1				
M3	0.57***	0.46***	1			
M4	0.1**	0.24***	0.27***	1		
M5	0.23***	0.45***	0.49***	0.3***	1	
M6	0.29***	0.31***	0.27***	0.17***	0.25***	1

Dependency			
	D1	D2	D3
D1	1		
D2	0.75***	1	
D3	−0.59***	−0.43***	1

Ethnic concentration		
	E1	E2
E1	1	
E2	0.46***	1

p value: *** <0.001; ** <0.01; * <0.05

### Spatial latent factor modelling

Factor loadings from the spatial latent factor model [Model (1)] are presented in Table 6. All indicators significantly load on their corresponding marginalization dimensions, with the expected positive or negative sign shown in Table 1. The posterior mean as well as the 95 % credible interval (CrI)^2^ of factor loadings ascertain indicators that most central to defining corresponding marginalization dimensions. For example, the level of material deprivation, dependency, and ethnic concentration seem to be mainly driven by the percentage of government transfer payment, percentage of seniors (65+), and percentage of visible minority, respectively, whereas all indicators of residential instability similarly relate to the constructed factor, with the exception of the percentage of residential mobility (same house as 5 years ago) which has a relatively low impact.

**Table 6:** Loadings of indicators on corresponding marginalization dimensions from Model (1)

ID	Indicator	Parameter	Posterior mean (95 % credible interval)
Loadings on residential instability			
1	% of living alone (R1)	δ1	1
2	% of youth population aged 5–15 (R2)	δ2	−0.984 (−1.081, −0.889)
3	Crowding: average number of persons per dwelling (R3)	δ3	−1.164 (−1.253, −1.078)
4	% of multi-unit housing (R4)	δ4	0.972 (0.872, 1.074)
5	% of the population that is married/common-law (R5)	δ5	−1.081 (−1.178, −0.987)
6	% of dwellings that are owned (R6)	δ6	−1.116 (−1.212, −1.025)
7	% of residential mobility (same house as 5 years ago) (R7)	δ7	0.491 (0.383, 0.604)
Loadings on material deprivation			
8	% 25+ without certificate, diploma, or degree (M1)	δ8	1
9	% of lone-parent families (M2)	δ9	0.747 (0.621, 0.875)
10	% of government transfer payment (M3)	δ10	1.194 (1.073, 1.319)
11	% of unemployment 15+ (M4)	δ11	0.313 (0.182, 0.447)
12	% of below low income cut-off (M5)	δ12	0.688 (0.559, 0.818)
13	% of homes needing major repair (M6)	δ13	0.738 (0.616, 0.862)
Loadings on dependency			
14	% of seniors (65+) (D1)	δ14	1
15	Dependency ratio [(0–14) + (65+)]/(15–64) (D2)	δ15	0.727 (0.597, 0.859)
16	Labor force participation rate (aged 15+) (D3)	δ16	−0.751 (−0.877, −0.629)
Loadings on ethnic concentration			
17	% of 5-year recent immigrants (E1)	δ17	1
18	% of visible minority (E2)	δ18	1.53 (1.352, 1.72)

**Fig. 3:**
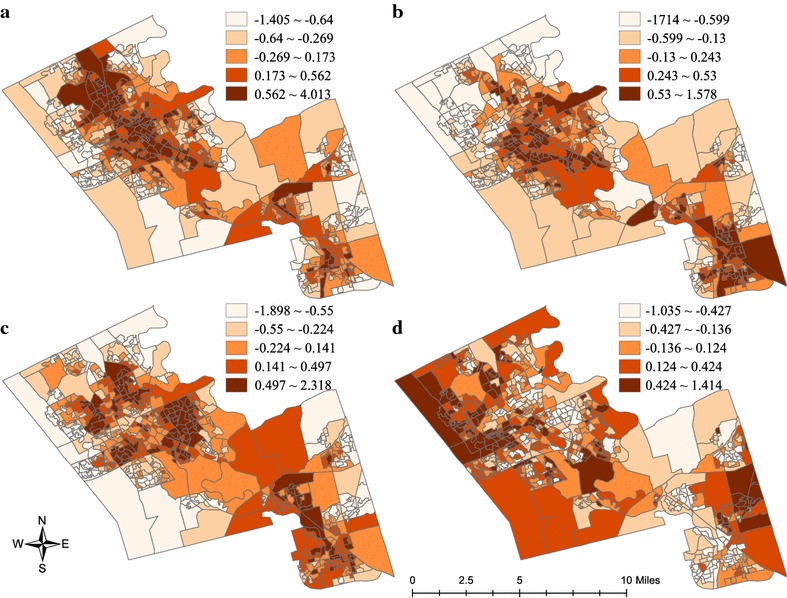
Quantile maps of marginalization dimensions at dissemination area scale, 2006. **a** Residential instability, **b** material deprivation, **c** dependency, **d** ethnic concentration

We map the four marginalization dimensions constructed from the spatial latent factor model (Fig. 3). Clear spatial patterns of the four marginalization dimensions can be identified from the map: areas with high residential instability locate along the main road—King Street—in the region, mainly concentrating in the central parts of Waterloo, Kitchener, and Cambridge. Highly materially deprived areas locate in central Waterloo, central and northeast Kitchener, and south Cambridge. Five distinct clusters of areas with high levels of dependency are found at south Waterloo, central Kitchener, and west Cambridge. As for areas with high ethnic concentration, they cluster at west Waterloo and east Cambridge, and scatter across the region.

### Spatial regression

Results regarding the associations between RFE healthfulness and marginalization dimensions as well as population density are presented in Table 7. The Bernoulli component of the spatial hurdle model [Model (2)] shows that residential instability (1.242, 95 % CrI 0.755–1.721), material deprivation (0.558, 95 % CrI 0.166–0.945), and population density (0.824, 95 % CrI 0.45–1.252) are significantly and positively associated with the probability of accessing healthy outlets within a walkable distance. These significant associations are not found in the binomial component [Model (3)]. Interestingly, a reversed direction is found between material deprivation and RFE healthfulness (−0.109, 95 % CrI −0.216 to −0.004). None of the marginalization dimensions or population density is found significantly related with RFE healthfulness with the 4 and 8 km datasets, with the exception of the negative association between dependency and RFE healthfulness at the 4 km scale (−0.022, 95 % CrI −0.042 to −0.002).

**Table 7:** Posterior estimates of coefficients from Models (2)–(4)

Covariate	Posterior mean (95 % credible interval)			
	1 km buffer		4 km buffer	8 km buffer
	Bernoulli	Binomial	Binomial	Binomial
Residential instability	*1.242* (*0.755*, *1.721*)	−0.004 (−0.088, 0.08)	−0.017 (−0.038, 0.005)	0.003 (−0.007, 0.013)
Material deprivation	*0.558* (*0.166*, *0.945*)	−*0.109* (−*0.216*, −*0.004*)	−0.018 (−0.042, 0.007)	−0.006 (−0.017, 0.006)
Dependency	0.168 (−0.227, 0.569)	0.019 (−0.067, 0.106)	−*0.022* (−*0.042*, −*0.002*)	−0.002 (−0.011, 0.008)
Ethnic concentration	−0.249 (−0.584, 0.074)	−0.02 (−0.101, 0.061)	0.006 (−0.013, 0.025)	−0.002 (−0.011, 0.007)
Population density	*0.824* (*0.45*, *1.252*)	−0.006 (−0.062, 0.05)	0.002 (−0.013, 0.016)	0.003 (−0.004, 0.011)

Significant coefficients are shown in italics

## Discussion

### Modelling results interpretations

In the region of Waterloo’s cities, neighbourhoods with higher residential instability and material deprivation are more likely to have access to healthy food outlets (i.e., better absolute healthy food access) within a walkable distance. This makes sense since healthy food outlets (Fig. 1) as well as residentially instable and materially deprived areas (Fig. 3) concentrate along the arterial streets of the region. This finding aligns with previous Canadian findings that socio-economically deprived residents have better access to absolute densities of healthy food outlets [10, 19, 22, 24–26, 30, 44, 58]. A probable explanation is that residents who are socio-economically deprived might be more likely to find affordable housing in highly populated areas [22, 24] where healthy food outlets are located, given that population density is a driving force of food outlet distribution as noted above.

In contrast, modelling the relative healthy food access (via binomial component from the spatial hurdle model), which represents RFE healthfulness in this study, reveals that areas with higher material deprivation have a relatively less healthy RFE at the walkable distance scale, despite higher probability to access to healthy food outlets. The finding is contrary to past Canadian studies that explored the relationship between material deprivation and relative healthy food access which is measured with crude proportions. For instance, most materially deprived neighbourhoods in Toronto were found to have healthier RFE (i.e., lower crude proportion of less healthy food outlets) [22]. Mercille et al. [58] reported that the poorest areas in Montreal have lower crude proportions of fast-food outlets over all accessible restaurants and higher crude proportions of fruit and vegetable stores over all accessible food stores in comparison to their wealthier counterparts. This inconsistency could be attributed to the differences between our research and previous studies in terms of the methods used for differentiating ‘healthy’ and ‘less healthy’ food outlets, the completeness of RFE datasets, and the appropriateness of statistical modelling approaches. Compared with the two Canadian studies noted above, our study is strengthened by differentiating ‘healthy’ and ‘less healthy’ based on in-store characteristics instead of food outlet types. This approach for defining healthy food outlets enabled all retail food outlets to be included in our dataset, which was a major strength of the current study. Also, in contrast to previous studies, we explicitly accounted for spatial autocorrelation occurring within RFE measures and marginalization indicators as demonstrated above, which increases the reliability of our results. Finally, we modelled the count of healthy food outlets with a binomial distribution rather than the crude proportion of healthy food outlets, which is associated with uncertainty thus is not a stable estimation of RFE ‘healthfulness’. As mentioned, our modelling approach is more robust to analyse relative healthy food access because it accounts for the underlying total number of accessible food outlets (thus the number of accessible less healthy food outlets) which cannot be reflected by crude proportions.

Not surprisingly, while increasing the buffering size to 4 km and 8 km (thus increased mobility) based on alternative transportation modes such as bicycling, public transit, and driving, population density and marginalization dimensions are not significantly associated with RFE healthfulness since discrepancies in relative healthy food access between areas decrease with larger travel distances (Fig. 2b, d, f). An exception is the negative association between dependency and RFE healthfulness at the 4 km scale, indicating that neighbourhoods with higher proportions of seniors and children have a less healthy RFE; however, this might not be problematic given that these dependent populations may be more likely to walk than to take public transit or bicycle.

### Policy implications

Findings from our study are important and informative for food environment planning and interventions for combating adverse diet-related health outcomes. Specifically, rather than improving absolute densities of healthy food outlets, a more pressing mission may be to strike a better balance between healthy and less healthy food access, especially given that increasing evidence shows that residents with higher relative healthy food access have healthier food purchasing [11, 59] and consumption [60, 61] behaviours, and lower body weight [62–65]. Traditional approaches such as building new supermarkets [66] have been proposed in the US for improving healthy food access thus the balance, but were found ineffective for promoting healthy eating [66] possibly due to residents’ hesitation of relying on a new food store [67].

Policy and program interventions to improve the food environment in Canada are nascent [68]. One potentially effective intervention for the Region of Waterloo could be modifying the in-store characteristics of existing food outlets in materially deprived areas, for example, providing fruits and vegetables in less healthy food outlets through intervention programs such as healthy corner stores, which have been implemented in municipalities of Toronto and Vancouver [69, 70]. This approach, if undertaken, should prioritize food outlets within a walkable distance to areas that fall inside the highest material deprivation quantile (Fig. 2b). An alternative intervention could be restricting the construction of less healthy food outlets within or around these neighbourhoods via zoning bylaws. While Canadian planning laws do not permit discrimination against specific types of food outlets [71, 72], the Regional Municipality of Waterloo can apply several urban planning tools to limit the establishment of less healthy food outlets, for example, prohibiting fast-food restaurant establishments and regulating the densities or quotas of less healthy food outlets in materially deprived neighbourhoods [71, 73].

Modelling results of 4- and 8 km-datasets suggest that increasing mobility might be effective for alleviating the disparities of RFE healthfulness. Yet travelling further to access healthier RFEs could economically burden materially deprived residents. As discussed in LeClair and Aksan [74], the high travelling costs might outweigh the cost savings from food shopping, thus deterring residents from taking public transit to procure healthy foods. In this sense, improving public transportation to healthy food retailers via interventions such as providing healthy food outlets (e.g., supermarkets) sponsored shuttle services could be potentially effective for encouraging materially deprived residents to travel beyond the walkable zones for food purchasing, complementary to aforementioned interventions.

### Methodology implications

Methodologically, this study contributes to the RFE literature by introducing a flexible modelling approach to study the association between neighbourhood RFE and marginalization. While the spatial lag [30] and spatial error [27, 30] models are inappropriate to model count data (e.g., number of supermarkets accessible to a DA), the applied Bayesian hierarchical approach can model the count of food outlets by following a discrete distribution, for example the binomial distribution as demonstrated in this study, while simultaneously account for spatial autocorrelation by including spatial random effects. Moreover, this Bayesian approach applied is superior to the spatial scan statistical method [29], which is also capable of modelling count data, in terms of its feasibility to incorporate covariates.

Another noticeable advantage of the applied Bayesian approach is its capability to model spatio-temporal RFE datasets, as demonstrated by Lamichhane et al. [32]. Future research could examine how neighbourhood RFE might change over time in tandem with varying levels of marginalization. Furthermore, the spatial hurdle model used for analysing the 1 km dataset accounts for zero-inflation, an issue rarely reported by past RFE studies but could occur in the case that a large portion of neighbourhoods in the study region have no access to (healthy) food outlets within a walkable distance. Not appropriately taking into account zero-inflation may result in biased or imprecise inferences. Although the negative binomial model implemented via conventional frequentist approaches can deal with zero-inflation in some cases, it cannot easily address the spatial autocorrelation issue.

### Study limitations

Findings of this study are subject to several limitations. First, to create the buffering zones, the geographic centroid rather than the population centroid was used to represent each DA. We consider this approach acceptable considering that most DAs are relatively small so geographic centroids approximate population centroids. Second, we used 1, 4, and 8 km to represent potential transportation modes; however, a unified travelling distance might not be suitable for all DAs. In reality, residents in different neighbourhoods could take different times to travel 4 km by bus due to varying public transit availability and routes. More nuanced methods for characterizing transportation-based RFE (see for example Farber et al. [75]) should be applied in future research. Lastly, ‘healthy’ and ‘less healthy’ were differentiated based on a binary category. Although we observed similar results by conducting sensitivity analysis with a more rigorous definition of healthy food outlets (i.e., outlets falling into the highest tercile instead of the highest two quartiles), this categorization approach should be refined in future studies.

## Conclusion

This paper contributes empirically and methodologically to the RFE literature that explores the association between neighbourhood marginalization and RFE healthfulness. Using Bayesian spatial hierarchical models, this research found that residents in neighbourhoods with higher residential instability, material deprivation, and population density are more likely to have absolute access to healthy food outlets within a walkable distance. Materially deprived neighbourhoods however, are also more likely to have a relatively less healthy RFE at the walkable distance scale. These findings indicate that a simple ‘yes’ or ‘no’ answer for the deprivation amplification hypothesis in the context of RFE is inappropriate. To infer a relatively unbiased conclusion, incorporating the complete RFE dataset, considering various assessment strategies (i.e., absolute and relative access) of RFE, and applying sound spatial statistical approaches are warranted.

For the Region of Waterloo in particular, striking the balance between healthy and less healthy food outlets in these neighbourhoods via interventions such as modifying in-store characteristics, restricting the opening of less healthy food outlets, and improving public transit to healthy food outlets may be warranted. The Bayesian spatial hierarchical modelling approach, including spatial latent factor and spatial hurdle models, as shown in this study can be further explored in other Canadian settings or different countries. Future research could tailor the buffering cut-offs for different types of food outlets, which are potentially linked to behaviours underlying travel patterns to visit specific types of food outlets and subtypes among them.

## Appendix 1: Formulation of a binomial hurdle model

A binomial hurdle model is a two-component mixture model that consists of a point mass at zero with a Bernoulli model accounting for the zero counts and a truncated binomial model accounting for the positive counts. The form of a binomial hurdle model is given in Model (5), where π_i_ is the probability that a DA has access to healthy food outlets; Y_i_ and N_i_ are the number of accessible healthy food outlets and total number of accessible food outlets of DA_i_, respectively; and p_i_ is the probability of a food outlet being healthy in DA_i_.

5 $$\Pr (\text{y} = \text{Y}_{\text{i}}) = \left\{{\begin{array}{*{20}l} {1 - \uppi_{\text{i}}} \hfill &\quad {(\text{Y}_{\text{i}} = 0)} \hfill \\ {\uppi_{\text{i}} \times \frac{{\text{Binomial}(\text{Y}_{\text{i}} |\text{p}_{\rm{i}},\text{N}_{\rm{i}})}}{{1 - \text{Binomial}(0|\text{p}_{\rm{i}},\text{N}_{\rm{i}})}}} \hfill &\quad {(\text{Y}_{\text{i}} > 0)} \hfill \\ \end{array}} \right.$$

## Appendix 2: Prior specification

### Priors for spatial latent factor model [Model (1)]

An improper flat prior *Uniform*(−∞, +∞) is specified to the intercept α_j_. To avoid the “flip-flop” problem [i.e., $\updelta_{\text{j}} \times \text{X}_{{\text{ni}}} = ( -\updelta_{\text{j}} ) \times ( - \text{X}_{{\text{ni}}} )$] and to achieve identifiability, we set δ_1_, δ_8_, δ_14_, δ_17_—the factor loading of the first indicator of corresponding marginalization dimensions—as one [46]. Alternatively, we can specify a prior distribution for these factor loadings to restrict their values to be positive [48, 77, 78]. A vague prior *Normal*(*0*, *1000*) is assigned to all other δ_j_’s. An intrinsic conditional autoregressive (ICAR) prior is assigned to marginalization dimensions X_ni_. Under this prior distribution, the expected mean of X_ni_ is the mean of X_n_’s in adjacent areas and the variance of X_n_, denoted as $\upsigma_{{\text{X}_{\text{n}} }}^{2} ,$ is inversely proportional to the number of adjacent areas to area i. Adjacency is defined as areas sharing at least one vertex, a common approach used in spatial analysis studies [55]. We use the same adjacency definition to specify priors to spatially structured parameters in Models (2)–(4). To address the identifiability issue between the scales of δ_j_ and X_ni_, we set the variance of **X**_**n**_ to 1, equivalent to standardizing **X**_**n**_ [79]. A non-informative prior *Gamma*(*0.5*, *0.0005*) is given to the reciprocal of the variance of indicator j, $\upsigma_{\text{j}}^{2} .$

### Priors for spatial hurdle model [Models (2), (3)]

An improper flat prior *Uniform*(−∞, +∞) is given to the intercepts α_1_ and α_2_. Regression coefficients **β**_**1**_ and **β**_**2**_ are specified with a vague prior *Normal*(*0*, *1000*). Considering the potential correlation between the binary and positive outcomes, for example, areas more likely to have access to healthy food outlets (higher π_i_) also have healthier RFE (higher p_i_), we specify multivariate distributions for the random effects. Specifically, the unstructured random effects were assumed to follow a bivariate normal distribution (u_1i_, u_2i_)^T^ ~ MVN(0, Ω) with means 0 and a 2 × 2 variance–covariance matrix Ω. A bivariate ICAR (BICAR) distribution is assigned to the spatial random effects such that ${\mathbf{s}}_{{\mathbf{i}}} = ({\text{s}}_{{1{\text{i}}}} ,{\text{s}}_{{2{\text{i}}}} )^{\text{T}} |{\mathbf{s}}_{{ - {\mathbf{i}}}} \sim{\text{BICAR}}\left( {\frac{1}{{{\text{n}}_{\text{i}} }}\sum\nolimits_{{{\text{j}} \in {\text{m}}_{\text{i}} }} {{\text{s}}_{\text{j}} ,\frac{1}{{{\text{n}}_{\text{i}} }}\sum } } \right),$ where n_i_ and m_i_ are the number and the set of adjacent areas of DA_i_, respectively, and again, Σ is a variance–covariance matrix. We specify an inverse Wishart prior with 2 degrees of freedom to Ω and Σ.

### Priors for spatial binomial model [Model (4)]

*Uniform*(−∞, +∞) and *Normal*(*0*, *1000*) are assigned to α and β, respectively. We give an ICAR prior with variance $\upsigma_{\text{s}}^{2}$ to the spatial random effect s_i_ and a prior of normal distribution with mean 0 and variance $\upsigma_{\text{u}}^{2}$ to the unstructured random effect u_i_. The reciprocals of $\upsigma_{\text{s}}^{2}$ and $\upsigma_{\text{u}}^{2}$ are further specified with a prior *Gamma*(*0.5*, *0.0005*).

## Abbreviations

CrI: credible interval
DA: dissemination area
DIC: deviance information criterion
NEMS-R: nutrition environment measures survey-restaurant
NEMS-S: nutrition environment measures survey-store
OLS: ordinary least square
RFE: retail food environment

## Footnotes

1 Marginalization occurs “when people are systematically excluded from meaningful participation in economic, social, political, cultural and other forms of human activity in their communities and thus are denied the opportunity to fulfil themselves as human beings” [76].

2 95 % credible interval is the range that there is 95 % probability that the posterior mean occurs.

## References

[1] Engler-Stringer R, Le H, Gerrard A, Muhajarine N (2014). The community and consumer food environment and children’s diet: a systematic review. BMC Public Health.

[2] Black C, Moon G, Baird J (2014). Dietary inequalities: what is the evidence for the effect of the neighbourhood food environment?. Health Place.

[3] Morland KB (2015). Local food environments: food access in America.

[4] Caspi CE, Sorensen G, Subramanian SV, Kawachi I (2012). The local food environment and diet: a systematic review. Health Place.

[5] Kirkpatrick SI, Reedy J, Butler EN, Dodd KW, Subar AF, Thompson FE, McKinnon RA (2014). Dietary assessment in food environment research: a systematic review. Am J Prev Med.

[6] Macintyre S (2007). Deprivation amplification revisited; or, is it always true that poorer places have poorer access to resources for healthy diets and physical activity ?. Int J Behav Nutr Phys Act.

[7] Lytle LA (2009). Measuring the food environment: state of the science. Am J Prev Med.

[8] Larson NI, Story MT, Nelson MC (2009). Neighborhood environments. disparities in access to healthy foods in the U.S. Am J Prev Med.

[9] Beaulac J, Kristjansson E, Cummins S (2009). A systematic review of food deserts, 1966–2007. Prev Chronic Dis.

[10] Minaker LM, Shuh A, Olstad DL, Black JL, Engler-Stringer R, Mah CL (2016). Retail food environments research in Canada: a scoping review. Can J Public Health.

[11] Mason KE, Bentley RJ, Kavanagh AM (2013). Fruit and vegetable purchasing and the relative density of healthy and unhealthy food stores: evidence from an Australian multilevel study. J Epidemiol Community Health.

[12] Centers for Disease Control and Prevention. Census tract level state maps of the modified retail food environment index (mRFEI). ftp://ftp.cdc.gov/pub/Publications/dnpao/census-tract-level-state-maps-mrfei_TAG508.pdf. 2011. Accessed 21 Oct 2013.

[13] Franco M, Diez Roux AV, Glass TA, Caballero B, Brancati FL (2008). Neighborhood characteristics and availability of healthy foods in Baltimore. Am J Prev Med.

[14] Zenk SN, Schulz AJ, Israel BA, James SA, Bao S, Wilson ML (2006). Fruit and vegetable access differs by community racial composition and socioeconomic position in Detroit, Michigan. Ethn Dis.

[15] VernezMoudon A, Drewnowski A, Duncan GE, Hurvitz PM, Saelens BE, Scharnhorst E (2013). Characterizing the food environment: pitfalls and future directions. Public Health Nutr.

[16] Shishehbor MH, Litaker D (2006). Letter: socioeconomic status and mortality. Ann Intern Med.

[17] Matheson FI, Dunn JR, Smith KLW, Moineddin R, Glazier RH (2012). Development of the Canadian marginalization index: a new tool for the study of inequality. Can J Public Health.

[18] Larsen K, Gilliland J (2008). Mapping the evolution of “food deserts” in a Canadian city: supermarket accessibility in London, Ontario, 1961–2005. Int J Health Geogr.

[19] Apparicio P, Cloutier M-S, Shearmur R (2007). The case of Montréal’s missing food deserts: evaluation of accessibility to food supermarkets. Int J Health Geogr.

[20] Hogan JW, Tchernis R (2004). Bayesian factor analysis for spatially correlated data, with application to summarizing area-level material deprivation from census data. J Am Stat Assoc.

[21] Zadnik V, Reich BJ (2006). Analysis of the relationship between socioeconomic factors and stomach cancer incidence in Slovenia. Neoplasma.

[22] Polsky JY, Moineddin R, Glazier RH, Dunn JR, Booth GL (2014). Foodscapes of southern Ontario: neighbourhood deprivation and access to healthy and unhealthy food retail. Can J Public Health.

[23] Borrell C, Mari-Dell’Olmo M, Serral G, Martinez-Beneito M, Gotsens M (2010). Inequalities in mortality in small areas of eleven Spanish cities (the multicenter MEDEA project). Health Place.

[24] Black J, Carpiano R, Fleming S, Lauster N (2011). Exploring the distribution of food stores in British Columbia: associations with neighbourhood socio-demographic factors and urban form. Health Place.

[25] Daniel M, Kestens Y, Paquet C (2009). Demographic and urban form correlates of healthful and unhealthful food availability in Montréal, Canada. Can J Public Health.

[26] Smoyer-Tomic KE, Spence JC, Raine KD, Amrhein C, Cameron N, Yasenovskiy V, Cutumisu N, Hemphill E, Healy J (2008). The association between neighborhood socioeconomic status and exposure to supermarkets and fast food outlets. Health Place.

[27] McKenzie BS (2014). Access to supermarkets among poorer neighborhoods : a comparison of time and distance measures. Urban Geogr.

[28] Lamb KE, Thornton LE, Cerin E, Ball K (2015). Statistical approaches used to assess the equity of access to food outlets: a systematic review. AIMS Public Health.

[29] Baker EA, Schootman M, Barnidge E, Kelly C (2006). The role of race and poverty in access to foods that enable individuals to adhere to dietary guidelines. Prev Chronic Dis.

[30] Wang H, Tao L, Qiu F, Lu W (2016). The role of socio-economic status and spatial effects on fresh food access: two case studies in Canada. Appl Geogr.

[31] Lamichhane AP, Warren J, Puett R, Porter DE, Bottai M, Mayer-Davis EJ, Liese AD (2013). Spatial patterning of supermarkets and fast food outlets with respect to neighborhood characteristics. Health Place.

[32] Lamichhane AP, Warren JL, Peterson M, Rummo P, Gordon-Larsen P (2015). Spatial–temporal modeling of neighborhood sociodemographic characteristics and food stores. Am J Epidemiol.

[33] Glanz K, Sallis JF, Saelens BE, Frank LD (2007). Nutrition environment measures survey in stores (NEMS-S): development and evaluation. Am J Prev Med.

[34] Saelens BE, Glanz K, Sallis JF, Frank LD (2007). Nutrition environment measures study in restaurants (NEMS-R): development and evaluation. Am J Prev Med.

[35] Nethery RC, Warren JL, Herring AH, Moore KAB, Evenson KR, Diez-Roux AV (2015). A common spatial factor analysis model for measured neighborhood-level characteristics: the multi-ethnic study of atherosclerosis. Health Place.

[36] Statistics Canada. Dissemination area (DA). 2016. http://www.statcan.gc.ca/start-debut-eng.html. Accessed 15 Apr 2016.

[37] Curtis JE, Grabb EG, Guppy N (2004). Social inequality in Canada: patterns, problems, and policies.

[38] MacLeod CM, Eisenberg A, Green DA, Kesselman JR (2006). The normative dimensions of equality. Dimensions of inequality in Canada.

[39] Townsend P, Phillimore P, Beattie A (1988). Health and deprivation: inequality and the north.

[40] Atkinson J, Salmond C, Crampton P. NZDep2013 index of deprivation. Wellington; University of Otago: 2014. http://www.otago.ac.nz/wellington/otago069936.pdf.

[41] Pampalon R, Hamel D, Gamache P, Philibert MD, Raymond G, Simpson A (2012). An area-based material and social deprivation index for public health in Quebec and Canada. Can J Public Health.

[42] Minaker LM, Raine KD, Wild TC, Nykiforuk CIJ, Thompson ME, Frank LD (2013). Objective food environments and health outcomes. Am J Prev Med.

[43] Minaker LM, Raine KD, Wild TC, Nykiforuk CIJ, Thompson ME, Frank LD (2014). Construct validation of 4 food-environment assessment methods: adapting a multitrait–multimethod matrix approach for environmental measures. Am J Epidemiol.

[44] Smoyer-Tomic KE, Spence JC, Amrhein C (2006). Food deserts in the prairies? Supermarket accessibility and neighborhood need in Edmonton, Canada. Prof Geogr.

[45] Luan H, Law J, Quick M (2015). Identifying food deserts and swamps based on relative healthy food access: a spatio-temporal Bayesian approach. Int J Health Geogr.

[46] Congdon P (2011). The spatial pattern of suicide in the US in relation to deprivation, fragmentation and rurality. Urban Stud.

[47] Congdon P (2008). The need for psychiatric care in England: a spatial factor methodology. J Geogr Syst.

[48] Congdon P (2016). Explaining variations in obesity and inactivity between US metropolitan areas. GeoJournal.

[49] Neelon B, Chang HH, Ling Q, Hastings NS. Spatiotemporal hurdle models for zero-inflated count data: exploring trends in emergency department visits. Stat Methods Med Res. 2014;1–19. doi:10.1177/0962280214527079 10.1177/0962280214527079 24682266

[50] Neelon B, Ghosh P, Loebs PF (2013). A spatial Poisson hurdle model for exploring geographic variation in emergency department visits. J R Stat Soc Ser A Stat Soc.

[51] Kazembe LN (2013). A Bayesian two part model applied to analyze risk factors of adult mortality with application to data from Namibia. PLoS One.

[52] Amek N, Bayoh N, Hamel M, Lindblade KA, Gimnig J, Laserson KF, Slutsker L, Smith T, Vounatsou P (2011). Spatio-temporal modeling of sparse geostatistical malaria sporozoite rate data using a zero inflated binomial model. Spat Spatiotemporal Epidemiol.

[53] Chen H-J, Wang Y (2014). The changing food outlet distributions and local contextual factors in the United States. BMC Public Health.

[54] Zenk SN, Schulz AJ, Israel BA, James SA, Bao S, Wilson ML (2005). Neighborhood racial composition, neighborhood poverty, and the spatial accessibility of supermarkets in metropolitan Detroit. Am J Public Health.

[55] Haining R, Law J, Griffith D (2009). Modelling small area counts in the presence of overdispersion and spatial autocorrelation. Comput Stat Data Anal.

[56] MRC Biostatistics Unit. WinBUGS. 2015. http://www.mrc-bsu.cam.ac.uk/software/bugs/the-bugs-project-winbugs/. Accessed 1 July 2015.

[57] Spiegelhalter DJ, Best NG, Carlin BP, van der Linde A (2002). Bayesian measures of model complexity and fit. J R Stat Soc Ser B Stat Methodol.

[58] Mercille G, Richard L, Gauvin L, Kestens Y, Payette H, Daniel M (2013). Comparison of two indices of availability of fruits/vegetable and fast food outlets. J Urban Health.

[59] Thornton LE, Bentley RJ, Kavanagh AM (2009). Fast food purchasing and access to fast food restaurants: a multilevel analysis of VicLANES. Int J Behav Nutr Phys Act.

[60] Clary CM, Ramos Y, Shareck M, Kestens Y (2015). Should we use absolute or relative measures when assessing foodscape exposure in relation to fruit and vegetable intake? Evidence from a wide-scale Canadian study. Prev Med.

[61] Mercille G, Richard L, Gauvin L, Kestens Y, Shatenstein B, Daniel M, Payette H (2012). Associations between residential food environment and dietary patterns in urban-dwelling older adults: results from the VoisiNuAge study. Public Health Nutr.

[62] Polsky JY, Moineddin R, Dunn JR, Glazier RH, Booth GL (2016). Absolute and relative densities of fast-food versus other restaurants in relation to weight status: does restaurant mix matter?. Prev Med.

[63] Mehta NK, Chang VW (2008). Weight Status and Restaurant Availability. A multilevel analysis. Am J Prev Med.

[64] Kestens Y, Lebel A, Chaix B, Clary C, Daniel M, Pampalon R, Theriault M, Subramanian SV (2012). Association between activity space exposure to food establishments and individual risk of overweight. PLoS One.

[65] Spence JC, Cutumisu N, Edwards J, Raine KD, Smoyer-Tomic K (2009). Relation between local food environments and obesity among adults. BMC Public Health.

[66] Cummins S, Flint E, Matthews SA (2014). New neighborhood grocery store increased awareness of food access but did not alter dietary habits or obesity. Health Aff.

[67] Morland KB, Morland KB (2015). Geography of local food environments: people and places. Local food environments: food access in America.

[68] Mah CL, Cook B, Rideout K, Minaker LM (2016). Policy options for healthier retail food environments in city-regions. Can J Public Health.

[69] Seeton M. Enhancing healthy food retail : models for increasing access to healthy local food in Vancouver neighbourhoods. 2012. https://sustain.ubc.ca/sites/sustain.ubc.ca/files/Local%20Food%20-%20Meredith%20Seeton%20-%20Healthy%20Food%20Retail%20Models.pdf. Accessed 10 Oct 2014.

[70] Toronto Food Policy Council. Food strategy update: healthy corner store project. 2014. http://tfpc.to/toronto-food/food-strategy-update-healthy-corner-store-project. Accessed 10 Sep 2015.

[71] Association Pour La Sante Publique Du Quebec (ASPQ). The school zone and nutrition: courses of action for the municipal sector. 2011. http://www.aspq.org/documents/file/guide-zonage-version-finale-anglaise.pdf. Accessed 20 May 2016.

[72] Grant JL, MacKay KC, Manuel PM, McHugh TLF (2010). Barriers to optimizing investments in the built environment to reduce youth obesity: policy-maker perspectives. Can J Public Health.

[73] Canadian Institute of Planners (CIP). Healthy communities practice guide. 2013. https://cip-icu.ca/Files/Healthy-Communities/CIP-Healthy-Communities-Practice-Guide_FINAL_lowre.aspx. Accessed 21 Nov 2015.

[74] LeClair MS, Aksan AM (2014). Redefining the food desert: combining GIS with direct observation to measure food access. Agric Hum Values.

[75] Farber S, Morang MZ, Widener MJ (2014). Temporal variability in transit-based accessibility to supermarkets. Appl Geogr.

[76] Rao DB (2007). Education for all: issues and trends.

[77] Abellan JJ, Fecht D, Best N, Richardson S, Briggs DJ (2007). Bayesian analysis of the multivariate geographical distribution of the socio-economic environment in England. Environmetrics.

[78] Marí-Dell’Olmo M M, Martínez-Beneito MA, Borrell C, Zurriaga O, Nolasco A, Domínguez-Berjón MF (2011). Bayesian factor analysis to calculate a deprivation index and its uncertainty. Epidemiology.

[79] Skrondal A, Rabe-Hesketh S (2007). Latent variable modelling: a survey. Scand J Stat.

